# Healthy human skin Kelvin-Voigt fractional and spring-pot biomarkers reconstruction using torsional wave elastography

**DOI:** 10.1007/s13246-024-01387-z

**Published:** 2024-02-06

**Authors:** Yousef Almashakbeh, Hirad Shamimi, Inas H. Faris, José M. Cortés, Antonio Callejas, Guillermo Rus

**Affiliations:** 1 Department of Structural Mechanics, University of Granada, 18071 Granada, Spain; 2 Instituto de Investigación Biosanitaria, ibs.GRANADA, 18012 Granada, Spain; 3 Excellence Research Unit,“Modelling Nature” (MNat), University of Granada, 18071 Granada, Spain

**Keywords:** Skin tissue reconstruction, Torsional wave elastography, Probabilistic inverse problem, Fractional rheological models, The spring pot model, The Kelvin-Voigt fractional model

## Abstract

This paper presents a novel method for reconstructing skin parameters using Probabilistic Inverse Problem (PIP) techniques and Torsional Wave Elastography (TWE) rheological modeling. A comprehensive examination was conducted to compare and analyze the theoretical, time-of-flight (TOF), and full-signal waveform (FSW) approaches. The objective was the identification of the most effective method for the estimation of mechanical parameters. Initially, the most appropriate rheological model for the simulation of skin tissue behavior was determined through the application and comparison of two models, spring pot (SP) and Kevin Voigt fractional derivative (KVFD). A numerical model was developed using the chosen rheological models. The collection of experimental data from 15 volunteers utilizing a TWE sensor was crucial for obtaining significant information for the reconstruction process. The study sample consisted of five male and ten female subjects ranging in age from 25 to 60 years. The procedure was performed on the ventral forearm region of the participants. The process of reconstructing skin tissue parameters was carried out using PIP techniques. The experimental findings were compared with the numerical results. The three methods considered (theoretical, TOF, FSW) have been used. The efficacy of TOF and FSW was then compared with theoretical method. The findings of the study demonstrate that the FSW and TOF techniques successfully reconstructed the parameters of the skin tissue in all of the models. The SP model’s the skin tissue $\eta$ values ranged from 8 to 12 $Pa \cdot s$, as indicated by the TOF reconstruction parameters. $\eta$ values found by the KVFD model ranged from 4.1 to 9.3 $Pa \cdot s$. The $\mu$ values generated by the KVFD model range between 0.61 and 96.86 kPa. However, FSW parameters reveal that skin tissue $\eta$ values for the SP model ranged from 7.8 to 12 $Pa \cdot s$. The KVFD model determined $\eta$ values between 6.3 and 9.5 $Pa \cdot s$. The KVFD model presents $\mu$ values ranging between 26.02 and 122.19 kPa. It is shown that the rheological model that best describes the nature of the skin is the SP model and its simplicity as it requires only two parameters, in contrast to the three parameters required by the KVFD model. Therefore, this work provides a valuable addition to the area of dermatology, with possible implications for clinical practice.

## Introduction

The assessment of skin’s mechanical condition can serve as a clinical marker for connective tissue disorders, such as Ehlers-Danlos syndrome and epidermolysis bullosa. These conditions are characterized by modifications in tissue functionality that are accompanied by structural alterations [1]. Understanding skin biomechanics is crucial when examining the skin as an independent organ. Nevertheless, precise skin models can also serve the purpose of providing insights for computational models of various skin-adjacent organs within the body, like biomechanical models of the breast [2].The most frequently utilized methods for measurement include suction [3, 4], torsion [5, 6], and traction [7]. The primary disadvantage of these methods is that they alter the skin’s natural tension state. This is because the experimental device must be attached to the epidermis for the duration of the test. As a result, it is extremely difficult to estimate and extract the preload value that mechanical devices induce, and this may have an impact on the measured values of the mechanical properties. Various elastographic methods have been used since the late 20th century to evaluate the stiffness of tissues. The quantitative calculation of shear stiffness is derived from the shear wave velocity, serving as an indicator of tissue stiffness. Both static (SE) and dynamic (DE) elastography methods have been developed to quantify tissue stiffness [8].

Novel methodologies using torsional vibrations, as explored in our study, are central to assessing the viscoelastic characteristics of soft tissues. The movement of torsional waves, described as elastic shear waves, occurs in a radial and deep manner within soft tissues, exhibiting a curved geometric trajectory. This fundamental understanding is critical for evaluating the mechanical functioning of various soft tissues. Valtorta et al. were pioneers in this field, suggesting a novel approach for quantifying the complex shear modulus of soft biological tissues using a torsional resonator. Their technique’s feasibility was demonstrated through *in vitro* research on bovine and pig liver [9]. Building on this groundwork, our study included the generation and detection of torsional waves using a novel technique, TWE, developed by our research team.TWE is emerging as a modern alternative to dynamic elastography. The primary objective is to establish mechanical biomarkers for assessing shear wave velocity and shear moduli in soft tissues [10]. The utilization of parameters derived from torsional waves as biomarkers has been further corroborated by Ouared et al., establishing their efficacy in analyzing the structural characteristics of soft tissues [11]. Moreover, Callejas et al. contributed by attempting the first validation of TWE using classical rheometry, employing the Verasonics Vantage system for this purpose, within the frequency range of 0.5 to 100 Hz [12]. This study also addresses some limitations noted in previous research. Notably, the use of Acoustic Radiation Force (ARF) was employed to overcome the smaller frequency range limitations of classical rheometry, comparing the outcomes of both methodologies within a similar frequency range, specifically 300 to 1000 Hz for TWE. Additionally, we reconstructed the Kelvin-Voigt (KV) viscoelastic characteristics in tissue-imitating hydrogel phantoms using a PIP technique, contrasting the TWE methodology outcomes with synthetic signals generated by the Finite-Difference Time-Domain (FDTD) method [13], which were evaluated at TWE frequencies of 1000 Hz. Contrastingly, Shear Wave Elastography (SWE) was examined across a broader frequency spectrum, from 300 to 2500 Hz. FDTD is commonly used to model physics phenomena in solid materials. consists of elastic wave propagation [14]. FDTD can simulate a wider frequency range and nonlinear material characteristics.

The application of fractional order dynamic models has been utilized in the examination of stress relaxation of arteries in an *in vitro * [15] and *in vivo * [16]. It is well known that a single spring pot can be decomposed into an infinite sequence of spring pots. [16]. This behavior facilitates the understanding of the connection between fractional-order dynamical models and the intricate, multi-scale physical composition of biological tissues, and thus fractional rheological models, such as the KVFD and SP models, have demonstrated their efficacy as robust tools for comprehensive and accurate simulation of biological tissues.

Inverse problems play a pivotal role in various scientific domains as they serve as a key mechanism for deducing causal elements based on observed data. [17]. Our primary focus is directed towards a specific category of inverse problems known as probability inverse problems. Probabilistic inverse problems pertain to a category of problems that involve the management of statistical uncertainty. This feature facilitates a more detailed interpretation of the data. Probabilistic methodologies provide as a means for resolving gaps between empirical findings and theoretical predictions, successfully accommodating the presence of uncertainties arising from disparities in model assumptions and errors in data measurements. [18, 19].

The objective of this study is to use PIP and experimental measurements obtained using TWE in order to reconstruct biomarkers within skin tissue. The present paper is structured into several distinct sections. Initially, a comprehensive examination of the primary material used for research is presented. The techniques employed are subsequently elucidated inside a dedicated section outlining the materials and methods. In order to determine the most suitable model for simulating the skin, both a KVFD model and a SP model with FDTD solutions were employed. The TWE probe is utilized for the purpose of collecting experimental information from a sample of 15 participants. The experimental data, along with the KVFD and SP models, are utilized in an inverse problem that reconstructs the viscoelastic characteristics of the volunteers. The text culminates with a comprehensive analysis and concise recapitulation.

## Materials and methods

**Fig. 1:**
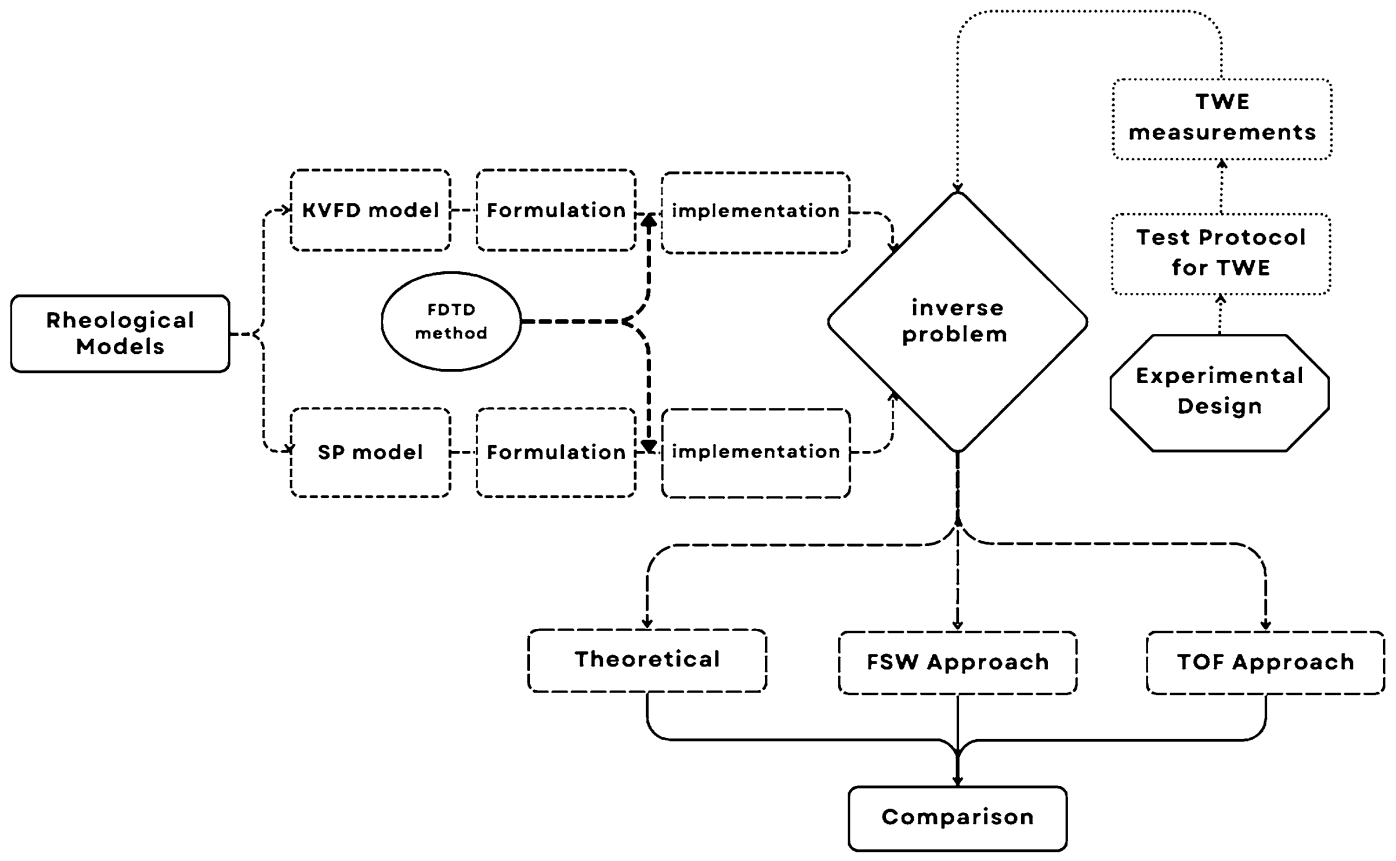
Flowchart for skin tissue biomarker reconstruction using torsional wave elastography

The methodology described in this study comprises four distinct steps. Two models, KVFD and SP, are utilized. Following their setup, the FDTD method is applied. An inverse problem is then encountered, connecting these models with the TWE testing process, which encompasses measurements and design details. Finally, results from FSW and TOF are compared using a standardized method (see Fig. 1).

### Rheological models

Soft tissue can be modeled as a linear viscoelastic medium, and several models have already been studied [20].We propose that this conclusion is substantiated by a comprehensive history in biomechanics, encompassing the concept of an extended relaxation spectrum, data gathered from viscoelastic soft tissues across various times and frequencies, and the theoretical framework involving multiple relaxations [21]. The conclusion that fractional derivative models accurately reflect reality is drawn from all models that simulate the multi-scale nature of biological tissues [21]. Thus, the SP model and the KVFD are suggested. In this study, we examine the complex modulus $G$, essential in viscoelastic analysis. It is expressed as $G = G' + iG''$, combining the storage modulus $G'$ and the loss modulus $G''$. Here, $G'$ represents the material’s elastic behavior, while $G''$ reflects its viscous behavior, with $i$ indicating the imaginary unit.

#### Formulation of the KVFD model

The KVFD model is an extension of the KV model. A parallel Hookean spring and a dashpot with a fractional derivative make up the KVFD model. The first derivative of the strain with respect to time in the KV model corresponds to the stress in the dashpot. The stress in the dashpot is equal to the fractional derivative of the strain of order $\alpha$. Three parameters are included in this viscoelastic model: $\mu$, $\eta$ and $\alpha$. $\mu$ denotes the relaxed elastic constant, $\eta$ denotes the viscoelastic parameter, and $\alpha$ denotes the fractional derivative order. For the KVFD model, the following model describes complex shear modules.

1 $$\begin{aligned} G_{\text {KVFD}} = \mu + \eta \omega ^{\alpha } \cos \left( \frac{\alpha \pi }{2}\right) + i \eta \omega ^{\alpha } \sin \left( \frac{\alpha \pi }{2}\right) \end{aligned}$$

where $G$ is complex shear modules and $\alpha$ is a the fractional derivative order. The KVFD model balances model simplicity and experimental precision well. KVFD has acquired prominence for its ability to capture both elastic and time-dependent viscous mechanical behavior of soft materials with only three model parameters [22]. Earlier studies have demonstrated that the KVFD model can achieve quasi-static loading targets at millimeter to centimeter spatial scales. It can be used for a wide range of soft matter from macro to nanoscale [22, 23]. The model provides a good fit to a wide range of soft matter mechanical responses observed in the experimental record, which supports the confidence in the estimates of the model parameters [22–24].

#### Formulation of the SP model

The SP element exhibits behavior that falls in between that of a linear spring and that of a linear dashpot. Theoretically, this intermediate behavior is described by the stress being proportional to the fractional derivative of the strain., i.e., $\sigma \propto K^\alpha \epsilon$, where the derivative order $\alpha$ is a mechanical parameter referred to as the fractional parameter [25]. To the knowledge of the authors, all the applications of fractional viscoelastic models considered in the literature use a real number between 0 and 1 as the fractional parameter.

The following model is a description of complex shear modules for the SP model.

2 $$\begin{aligned} G_{\text {s.p}} = \eta \omega ^{\alpha } \cos \left( \frac{\alpha \pi }{2}\right) + i \eta \omega ^{\alpha } \sin \left( \frac{\alpha \pi }{2}\right) \end{aligned}$$

#### Shear velocity and the complex shear modulus for KVFD and SP

The dispersion shear wave velocity curve of each model has been calculated. It is possible to determine the viscoelastic tissue properties by comparing the data to a mechanical tissue model and using the curve as a reference. The terms “shear elasticity” and “shear viscosity” are represented in units of Pa and $Pa\cdot s$, respectively. The shear wave speed, $C_s(\omega )$, can be expressed as a function of frequency $\omega$. The storage modulus and loss modulus are denoted by $G'$ and $G''$, respectively, and the medium’s density is represented by $\rho$. This relationship is well established in the display of dispersion curves [26].

3 $$\begin{aligned} C_s(\omega ) = \sqrt{\frac{2(G'^2 + G''^2)}{\rho ((G + \sqrt{G'^2 + G''^2}))}} \end{aligned}$$

#### Equation derivation for KVFD model

According to recent studies, the KVFD constitutive law should be applied to depict the shear wave propagation in elastography. The researchers developed a numerical method based on FDTD to simulate compressional wave propagation in biological applications. This was done under the assumption of a KVFD constitutive law. The model was verified by showing good agreement with an analytical solution in a homogenous medium mimicking breast fatty tissue [27]. In order to mimic the propagation of shear waves produced by ARF in diverse viscoelastic materials, they also used a time-fractional wave equation. The authors utilized a time-to-peak technique similar to that used in SWE to assess the stiffness. The outcomes showed that the method worked well with lossless or low-loss media [28]. Using SWE, so-called Hyper-Frequency Viscoelastic Spectroscopy (HFVS), and a KVFD model, researchers successfully matched shear wave dispersion curves acquired from *in vitro * tests [29]. HFVS is an advanced rheological characterization technique that operates at frequencies up to 2 kHz. The model suggested in this paper was validated using a KVFD mode because it could accurately depict the absorption and dispersive effects according to the literature review findings. The equations of motion, the kinematic connection, and the constitutive equations were used to establish the equations regulating the propagation of torsional waves through the phantom for the KVFD model [30].

4 $$\begin{aligned} \frac{\partial }{\partial t} \left( \frac{\partial u_\theta }{\partial t} \right)= & {} \frac{\partial \sigma _{r\theta }}{\partial r} + \frac{\partial \sigma _{\theta z}}{\partial z} + \frac{2}{r} \sigma _{r\theta } \end{aligned}$$

5 $$\begin{aligned} \sigma _{r\theta }= & {} \left( \mu + \eta \left( \frac{\partial ^\alpha }{\partial t^\alpha }\right) \right) \left( \frac{\partial u_\theta }{\partial r} - \frac{u_\theta }{r} \right) \end{aligned}$$

6 $$\begin{aligned} \sigma _{\theta z}= & {} \left( \mu + \eta \left( \frac{\partial ^\alpha }{\partial t^\alpha }\right) \right) \left( \frac{\partial u_\theta }{\partial z} \right) \end{aligned}$$

where $\rho$ represents the phantom density, $\mu$, $\eta$ and $\alpha$ (KVFD parameters) denote the shear elasticity, viscosity, and order of the fractional derivative, respectively, r, $\theta$ and z denotes the cylindrical components, u denotes the particle displacement, and the $\sigma$ denotes the stress tensor. The standard components of the equation system were eliminated, leaving just the deviatoric (torsional) components. This simplification had no impact on the outcomes, According to the Orescanin et al. experiment, this is due in part to the reduced normal pressure generated by the exciter-in this case, the torsion probe [14].

#### Equation derivation for spring-pot model

**Fig. 2:**
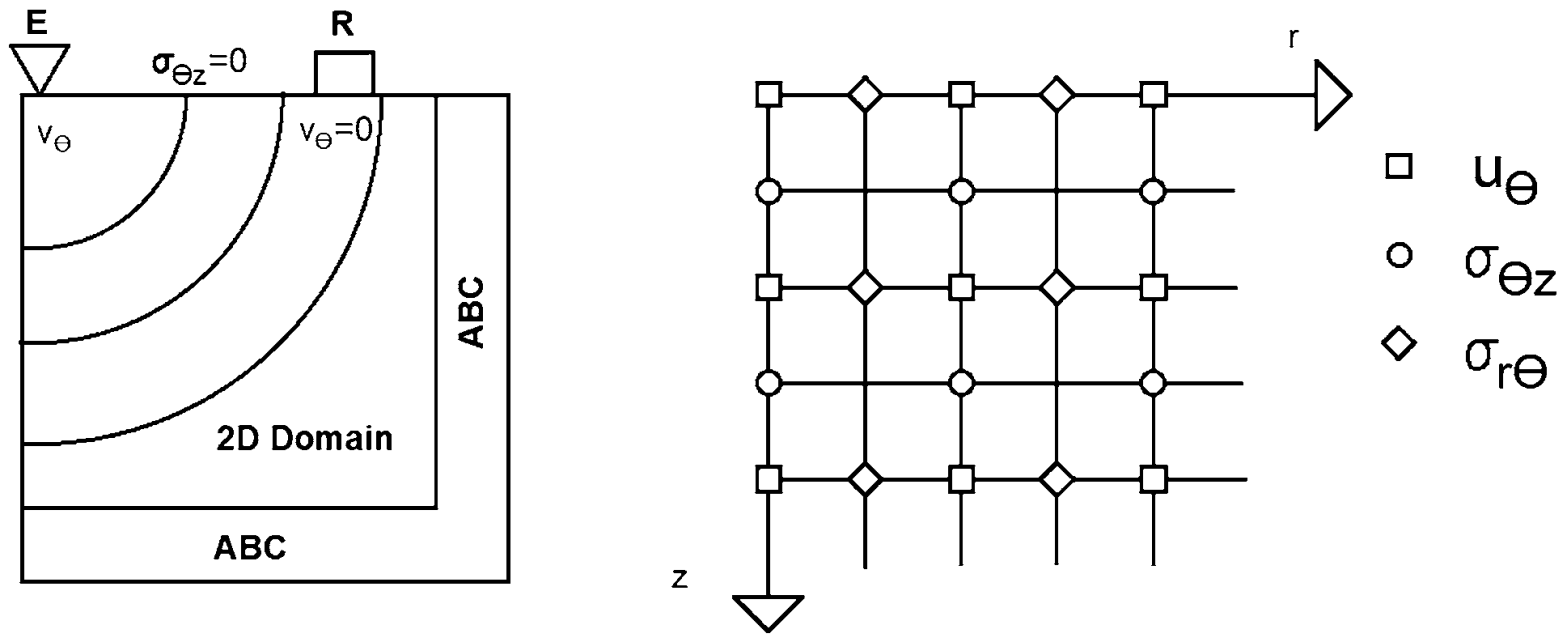
The spatial distribution of the model boundary conditions. A two-dimensional domain is bordered by absorbing boundary conditions, excitation, reception ($v_{\theta }$=0), and free surface conditions

The robust viscoelastic behavior of the materials in our study can be represented by power laws, which effectively capture a variety of experimental data in both the time and frequency domains [31]. The particles of a material vibrate perpendicular to the wave’s passage in a mechanical disturbance called a torque wave. As mechanical waves, they interact with tissue mechanics and their spatial variability. Recently, torsional waves were used to patent a new family of sensors. This concept is shown by low-frequency sensors that apply torsional shear waves in a largely solid medium [28]. Strong attenuation is a difficulty resolved in this new technology, which can generate shear waves of more considerable strength, allowing for novel noninvasive tissue characterization methods. The probe used in this investigation was made to allow quick measurements of the shear stiffness of the cervix. The excitation energy deposited in the tissue, the spurious waves (P-waves), and their reliance on the applied pressure sensor tissue have all been removed. This technique is similar to that described in [32]. The method is easy to use. It guarantees a simple clinical procedure and a short learning curve for the operator. This section describes creating a 2D numerical model for torsion wave propagation in skin tissue. A simpler skin-like substance was geometrically represented as a solid cylinder. Torsional waves propagate from the axisymmetrically of the sample center. [12]. A cylindrical coordinate system (r,$\theta$,z) can be employed because of the geometric layout. The expense of computing 3D models is considerable. As a result, while maintaining the majority of the physics, 2D simplifications are useful for exploratory study. This geometry was reduced to a two-dimensional instance using absolute axial symmetry. A TWE source generates an axisymmetrically propagating wavefront. Due to this characteristic and the axisymmetrical design of the model, the displacement field is reduced to just one element, the angular displacement $u_\theta$. All derivatives relating to the variable $\theta$ will also be zero.The two-dimensional domain for the wave propagation model was chosen to be the r–z plane (see Fig. 2). The equations of motion, the kinematic relationship, and the constitutive equations were used to obtain the equations regulating the propagation of torsional waves through the tissue for the SP model. All of the standard components have been eliminated from the equation system, retaining only the deviatoric (torsional) components. This simplification did not seem to have an impact on the results, according to Orescanin et al., because of the low normal pressure produced by the exciter in this case, the torsional probe [13, 14].

7 $$\begin{aligned} \frac{\partial }{\partial t} \left( \frac{\partial u_\theta }{\partial t}\right)= & {} \frac{\partial \sigma _{r\theta }}{\partial r} + \frac{\partial \sigma _{\theta z}}{\partial z} + \frac{2}{r} \sigma _{r\theta } \end{aligned}$$

8 $$\begin{aligned} \sigma _{r\theta }= & {} K_\alpha \frac{\partial ^\alpha }{\partial t^\alpha } \left( \frac{\partial u_\theta }{\partial r} - \frac{u_\theta }{r}\right) \end{aligned}$$

9 $$\begin{aligned} \sigma _{\theta z}= & {} K_\alpha \frac{\partial ^\alpha }{\partial t^\alpha } \left( \frac{\partial u_\theta }{\partial z}\right) \end{aligned}$$

### Numerical implementation

The FDTD method has been widely used to model various physical phenomena in solid materials. but also for wave propagation in elastic [14]. The FDTD solutions can represent a more comprehensive frequency range with a single simulation process and naturally treat nonlinear material properties [33, 34]. Nonetheless, numerous physics events in solid materials have been modeled using the FDTD approach for elastic wave propagation. FDTD solutions naturally handle nonlinear material features and can represent a more excellent frequency range with a single simulation session [14]the KVFD and SP equation system was solved using the FDTD method.

#### Implementation of KVFD model using FDTD method

The equations that describe the physical phenomenon of wave propagation in a KVFD viscoelastic medium are found in [30]. These equations are then transformed into discrete equations by applying space-time discretization to the domain and FD expressions to the equations. The propagation of shear waves was modeled using the FDTD method. The velocity stress formulation of Virieux has been modified by Gomez et al. to create their model. [35]. The number of temporal derivatives and related processing costs was reduced with this approach [30, 36].

#### Implementation of SP model using FDTD method

Discrete equations are produced by applying space-time discretization to the domain and FDTD expressions to the equations that describe the phenomenon of wave propagation in a spring-pot viscoelastic medium. The approach for incorporating the Perfect Matching Layer (PML) parameters for cylindrical coordinates was established by Liu [37]. First, the equations of the problem are separated. Expressions (10–12) compose the conservation of momentum equation in cylindrical coordinates. The SP constitutive law equations incorporate the strain–displacement relationship, which reduces the amount of memory required to calculate the approach. The resulting equations are then divided into the expressions (13–15) and differentiated within time.

10 $$\begin{aligned} a_r \frac{\partial ^2 u_\theta (r)}{\partial t^2} + \omega _r \frac{\partial u_\theta (r)}{\partial t}= & {} \frac{\partial \sigma _{r\theta }}{\partial r} \frac{1}{\rho } \frac{\partial r}{\partial r} \end{aligned}$$

11 $$\begin{aligned} A_r \frac{\partial ^2 u_\theta (\theta )}{\partial t^2} + \Omega _r \frac{\partial u_\theta (\theta )}{\partial t}= & {} \frac{2\sigma _{r\theta }}{\rho } \end{aligned}$$

12 $$\begin{aligned} a_z \frac{\partial ^2 u_\theta (z)}{\partial t^2} + \omega _z \frac{\partial u_\theta (z)}{\partial t}= & {} \frac{\partial \sigma _{\theta z}}{\partial z} \frac{1}{\rho } \frac{\partial z}{\partial z} \end{aligned}$$

13 $$\begin{aligned} a_r \frac{\partial \sigma _{r\theta }(r)}{\partial t} + \omega _r \sigma _{r\theta }(r)= & {} K_\alpha \frac{\partial ^{\alpha +1}}{\partial t^{\alpha +1}} \left( \frac{\partial u_\theta }{\partial r}\right) \end{aligned}$$

14 $$\begin{aligned} A_r \frac{\partial \sigma _{r\theta }(\theta )}{\partial t} + \Omega _r \sigma _{r\theta }(\theta )= & {} -K_\alpha \frac{\partial ^{\alpha +1} u_\theta }{\partial t^{\alpha +1}} \end{aligned}$$

15 $$\begin{aligned} a_z \frac{\partial \sigma _{\theta z}(z)}{\partial t} + \omega _z \sigma _{\theta z}(z)= & {} K_\alpha \frac{\partial ^{\alpha +1}}{\partial t^{\alpha +1}} \left( \frac{\partial u_\theta }{\partial z}\right) \end{aligned}$$

where $u_\theta = u_\theta (r) + u_\theta (\theta ) + u_\theta (z)$ according to the notation employed by [37]. $a_r$, $A_r$, $a_z$, $\omega _r$, $\Omega _r$, and $\omega _z$ are the PML variables described by Liu (1999). $a_r = a_z = 1$, while $A_r = \frac{1}{r}$. $\omega _r$, $\Omega _r$, and $\omega _z$ are the absorbing parameters.

#### Problem setup and research range

The size of typical human skin tissue was considered when choosing the dimension ranges. The skin thickness of males ranged from 0.6 mm to 3.3 mm, whereas females ranged from 1.3 mm to 3.1 mm [38]. The FDTD wave propagation model was implemented in MATLAB ®(Release, 2018a) (Release 2018a, MathWorks, Natick, United States) using the Parallel Computing Toolbox. There was no physical model of the torsion probe. Instead, the mesh components of the model surface where the probe would be placed were immediately exposed to the excitation displacement signal. Similarly, Fig. 2 shows that the displacement values at the mesh elements in direct physical contact with the positions of the sensors were recorded rather than modeling the array of sensors. A collection of absorbing components with an exponential attenuation factor is known as the ABCs. The attenuation act mimics an endless boundary condition by diminishing reflections. The dimensions of the emitter, receiver, emitter-receiver, and receiver-ABC are shown in Fig. 2. The following were the two-dimensional space border conditions (Fig. 2): The excitation source at specific locations on the sample surface was the problem boundary condition, as was the absence of shear stress on the surface as a free boundary. also, the absence of velocity in the grid point at the receiving location due to the pressure between the probe receiver and the sample. [13].

$$\begin{aligned} \sigma _{\theta z}(r,0,t_n) = 0 \\ v_\theta (r_{\text {reception}},0,t_n) = 0 \end{aligned}$$

### Experimental procedure: in vivo torsional wave elastography on healthy human skin

The research procedures and methods used in this study have been rigorously evaluated and approved by the Bioethics Committee of the San Cecilio University Clinical Hospital, with reference number 0824-N-22. In order to acquire a comprehensive understanding of our newly developed TWE device, a series of systematic experiments were conducted.

**Fig. 3:**
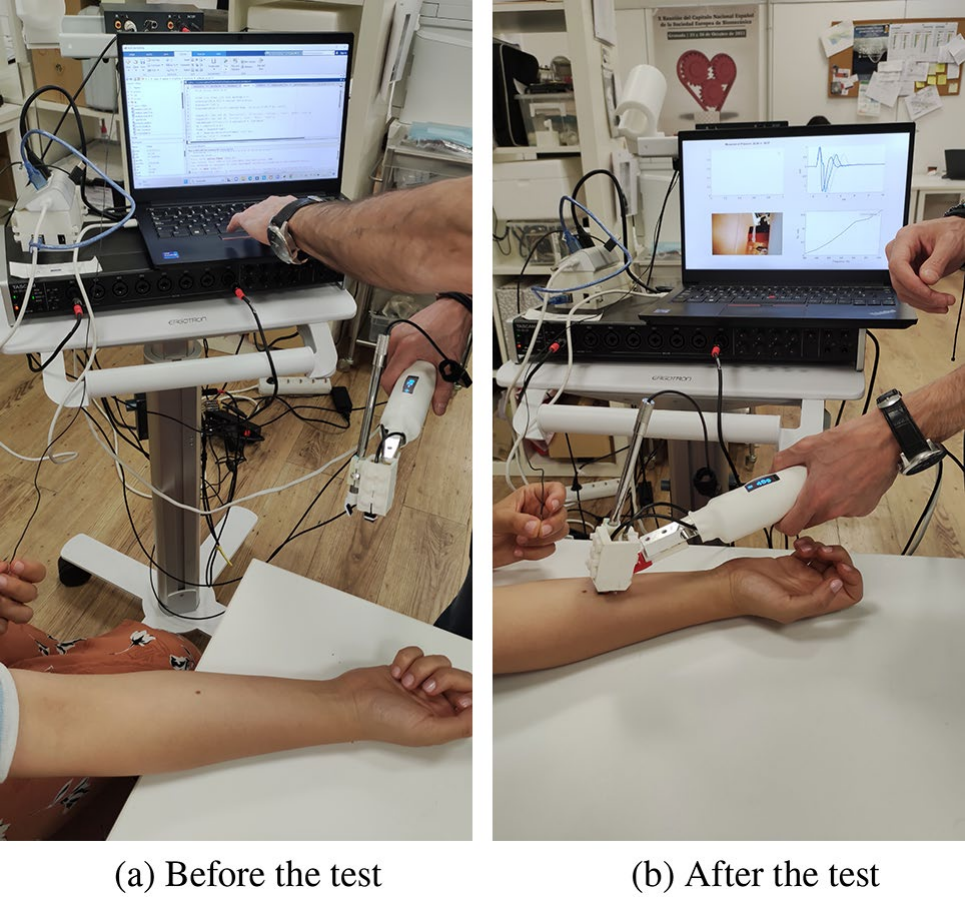
TWE measurement setup showing the participant’s forearm with the internally developed TWE device, comprising an emitter (generating shear waves within 0.1 to 2 kHz) and a receiver (3 mm from the emitter), aligned parallel and transverse to the Langer lines

Figure 3 depicts the experimental setup and procedures employed for the TW sensor. The operation was conducted on the ventral forearm region of a cohort of individuals who willingly consented to participate in our research investigation. The study’s sample size consisted of 15 participants, who were carefully selected through a rigorous interview process to enhance the reliability and applicability of the findings. In order to accomplish this, the study included a total of fifteen people, with five identifying as male and ten as female. The age range of the participants spanned from 25 to 60 years, and it was ensured that all participants had no prior record of skin disease.

The TWE devices developed internally consist of two components, namely an emitter and a receiver. The emitter possesses the ability to generate shear elastic waves within a frequency range of 0.1 to 2 kHz. The receiver, positioned at a distance of 3 mm from the emitter, effectively captures the propagating waves [8, 12, 13]. The testing procedure was developed with the inclusion of a frequency range spanning from 0.4 to 1 kHz. The selection of this particular range was based on its ability to facilitate the extraction of essential biomarkers, including skin tissue viscosity and stiffness, which play a significant role in assessing the overall well-being and state of the skin. To ensure uniformity and minimize any variability during the testing method, specific protocols were devised. The participant’s forearm was positioned on a level surface, and they maintained a state of relaxation during the entirety of the the procedure. The probe was positioned in a parallel and transverse manner with respect to the Langer lines.

During the experimental procedure, a consistent pressure of 30 gs was uniformly exerted on all participants. The maintenance of consistency played a crucial role in mitigating discrepancies in the outcomes produced and maintaining control over the entirety of the testing procedure. The selection of this specific pressure value was conducted with careful consideration, as it was determined to yield legible outcomes while ensuring the safety and well-being of the participating volunteer by avoiding any potential harm or unnecessary pain.

### Reconstructing the mechanical characteristics of skin tissue using a probabilistic inverse problem

Viscoelastic parameters are reconstructed based on the influence of viscoelasticity on the wave velocity in soft tissue. Because the cause can be inferred from the effect in this situation, our problem is an inversion of a forward problem. Our primary focus is directed towards a specific category of inverse problems known as PIP. In our study, we employed the PIP method, crucial for discerning underlying causes from observable effects in viscoelastic material characterization. This is vital where direct observation of parameters is challenging, addressing the inverse problem to determine initial conditions or causes. Our methodology’s strength lies in handling noise-contaminated data using Bayesian techniques. This enables identifying the most plausible model satisfying the maximum likelihood criterion, enhanced by prior information on model parameters. We introduced two informational sources: $f_o$ from experimental observation and $f_m$ from the numerical model, treated concurrently:

16 $$\begin{aligned} \begin{aligned} f(O, M, H)&= f(O, M, H)^O \text { and } f(O, M, H)^m \\&= f(O, M, H)^O f(O, M, H)^m \end{aligned} \end{aligned}$$

The parametrization process within the information-theoretic inverse problem framework is:

17 $$\begin{aligned} f(M)_{H=H_K} = k_1 \int _{o} f^o(O) f^o(M) f^M(OMH) \, dO \end{aligned}$$

For model parametrization, we used:

18 $$\begin{aligned} {\tilde{m}}_i = \frac{\ln \left( \frac{m_i}{m_{i}^{\text {inf}}} \right) }{\ln \left( \frac{m_{i}^{\text {sup}}}{m_{i}^{\text {inf}}} \right) } \end{aligned}$$

Probability densities for observational and model data are assessed by:

19 $$\begin{aligned} \begin{aligned} f^o(o_i(t))&= k_2 \exp \left( -\frac{1}{2} \int \sum _{i,j=1}^{N_i} \left( o_i(t) - o_i^o(t) \right) \left( C_{ij}^o \right) ^{-1} \right. \\&\left. \quad \left( o_i(t) - o_i^o(t) \right) \, dt \right) \end{aligned} \end{aligned}$$

20 $$\begin{aligned} \begin{aligned} f^m(o_i(t), M, H_K)&= k_3 \exp \left( -\frac{1}{2} \int \sum _{i,j=1}^{N_i} \left( o_i(t) - o_i(t, M, H_K) \right) \left( C_{ij}^m \right) ^{-1} \right. \\&\left. \quad \left( o_i(t) - o_i(t, M, H_K) \right) \, dt \right) \end{aligned} \end{aligned}$$

21 $$\begin{aligned} \begin{aligned} f(M)_{H=H_K}&= k_4 \exp \left( -\frac{1}{2} \int \sum _{i,j=1}^{N_i} \left( o_i(t, M, H_K) - o_i^o(t) \right) \right. \\&\left. \quad \left( C_{ij}^o + C_{ij}^m \right) ^{-1} \left( o_i(t, M, H_K) - o_i^o(t) \right) \, dt \right) \end{aligned} \end{aligned}$$

This framework effectively applies the PIP approach, determining the most probable parameters for our viscoelastic model and offering robust analysis of soft tissue mechanical properties [39–48]. The parameter search range values have been established using scientific evidence.

#### TOF approach for parameter reconstruction

The acquisition of the emitter and receiver signals is required for computational simulations. Through the computation of a cross-correlation function, R($\tau$), the relationship between these signals is quantified. This function assists in identifying the TOF by enabling to identify the time lag, $\tau$, that corresponds to its highest value [49]. The dispersion curve is then determined by conducting a frequency variation in the 400-1000 Hz range once the wave velocity has been established. This procedural paradigm is used to compare two different models, resulting in better comparison. Acquisition of velocity values for seven distinct frequencies per study participant is required for the collection of data from experiments. The resolution of inverse problems, the last step, represents an optimization problem. The difference between computational and experimental velocities is measured by the cost function inside a specific parameter space. The most effective parameters that minimize this discrepancy are thus provided by the optimization process. When considered as a whole, these steps offer an accurate and reliable base for parameter reconstruction utilizing the TOF methodology.

#### Full signal wave approach for parameter reconstruction

The complete waveform signal from the receiver is subjected to a detailed analysis during the experimental phase. We determine which simulated signal matches the experimental one using an inverse problem-solving strategy and an established best set of parameters. The average signal calculation:

22 $$\begin{aligned} \text {average of signal} = \frac{1}{N} \sum _{i=1}^{N} s_i \end{aligned}$$

where $s_i$ is the amplitude of the signal at a certain time and *N* is the number of data points utilized to determine the signal’s mean. Time-Frequency Representation and Pearson correlation are the analytical methods used in this process. Time-Frequency Signal representation highlights non-stationary properties by transforming the signal into the time-frequency domain [50], Pearson correlation quantifies linear interdependence. Collectively, these approaches enable it to be more straightforward to reconstruct accurate parameters and improve the precision of system parameter estimation.

## Results

### Experimental results

**Fig. 4:**
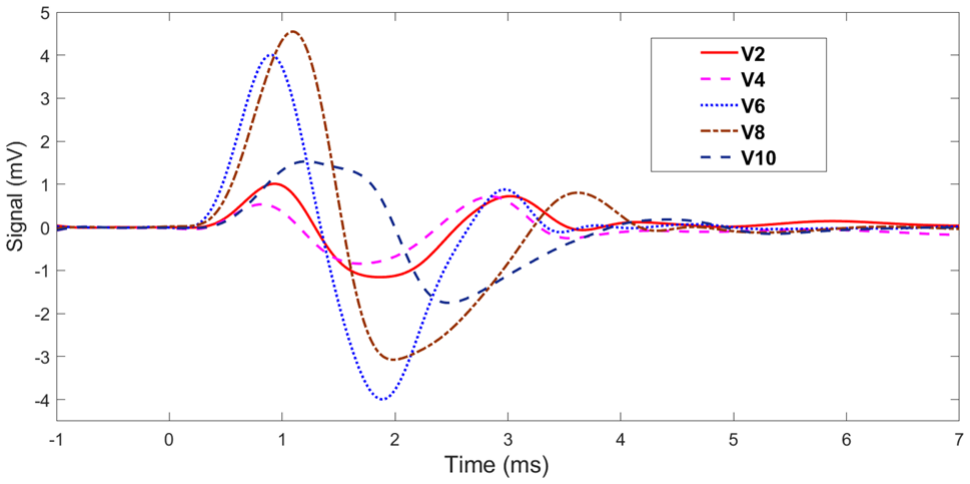
Examples of experimental signals at 500 Hz for volunteer 2, 4, 6, 8 and 10

Various experimental signals generated by the TWE sensor designed and fabricated by the Ultrasonics Lab team are depicted in Fig. 4. The signals shown are data from tests conducted on a sample of volunteers at a frequency of 500 Hz. The PIP utilized these signals to extrapolate tissue properties, which will be elaborated in the subsequent section. Table 1 shows the velocities measured at 400 Hz for volunteers using the TWE sensor. The TWE sensor’s data were analyzed using the TOF method to produce shear wave velocity. Calculation of the time offsets between the excitation and reception signals for each volunteer was essential to this investigation. Table 2 shows the skin tissue parameters reconstructed from the shear wave velocities obtained by analyzing the TWE data as a function of frequency using Equations 4 and 5. Finally, in Fig. 5, we show the dispersion curves for volunteers 2, 4, 6, 8, and 10. This frequency domain analysis focuses on the frequency range 400 to 1000 Hz.

**Table 1:** The measurements of the velocity at 400 Hz for the volunteers using the TWE sensor

# of Volunteer	Velocity (m/s)
V1	6.62
V2	6.27
V3	5.75
V4	6.53
V5	5.61
V6	7.01
V7	5.89
V8	5.24
V9	7.01
V10	4.64
V11	6.04
V12	5.96
V13	5.75
V14	5.48
V15	5.82

**Table 2:** Reconstruction of the parameters of the healthy skin using the theoretical method for the Spring-Pot and KVFD models

# of V	SP model		KVFD model		
	$\varvec{\eta }$ $(Pa \cdot s)$	$\varvec{\alpha }$	$\varvec{\eta }$ $(Pa \cdot s)$	$\varvec{\alpha }$	$\varvec{\mu }$ (Pa)
1	8	0.95	9.3	0.95	16070
2	11.5	0.9	8.1	0.95	18605
3	7.5	0.95	5.6	1	4110
4	7.5	0.95	9.3	0.95	15385
5	9.5	0.9	4.1	1	18740
6	11	0.95	7.3	1	1440
7	9.8	0.9	6.1	0.95	20840
8	11.3	0.9	5.1	1	6525
9	11.5	0.9	4.1	1	30070
10	10.8	0.85	3.2	1	9830
11	11	0.95	7.1	1	1585
12	11	1	5.6	1	4930
13	8.3	0.95	5.3	1	4315
14	8.3	0.95	4.9	1	4005
15	10.5	0.9	6.1	0.95	24415

**Fig. 5:**
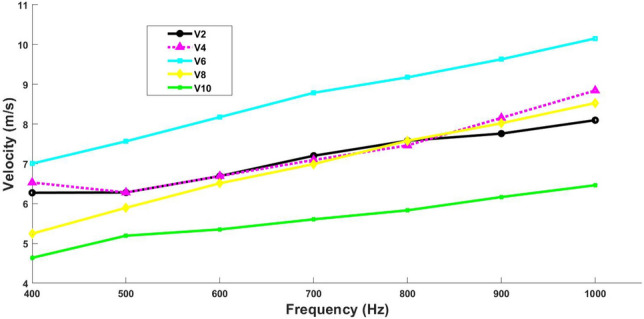
Examples of dispersion curves for volunteers 2, 4, 6, 8, and 10

### Reconstruction of parameters using the TOF and FWS methods

**Fig. 6:**
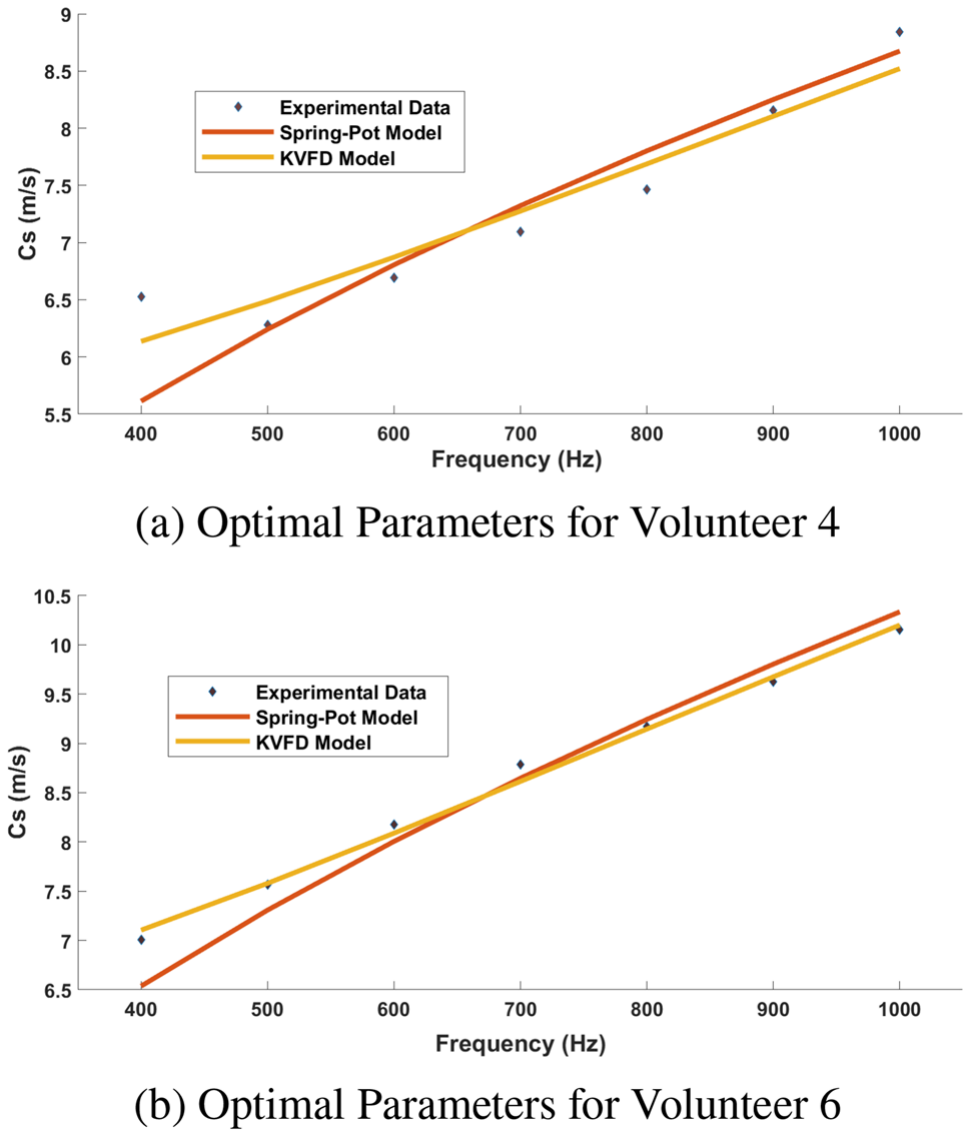
Dispersion Curves of Optimal Parameters for Volunteers 4 and 6 in the Spring-Pot and KVFD Models

**Fig. 7:**
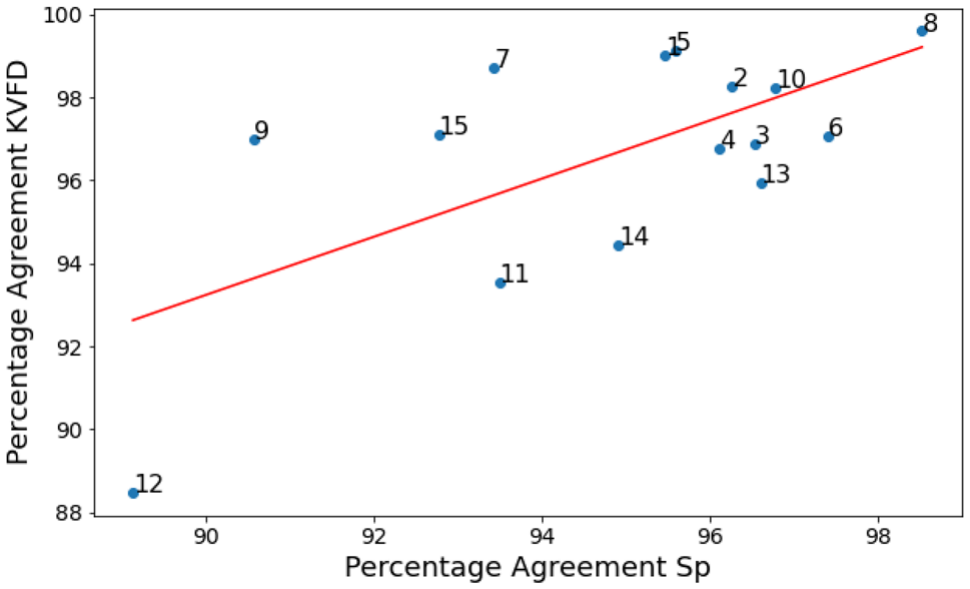
Quantitative Assessment of Model Accuracy: Percentage Agreement Between Experimental TOF and Predictions by SP and KVFD Models Using TWE Sensor

Figure 6 shows the experimental data acquired with the TWE sensor to the dispersion curves of the optimum parameters, obtained via the PIP, for volunteers 4 and 6. The level of agreement between the TOF in each model and the TWE sensor for all participants was quantified and is shown in Fig. 7 and Table 3. The percentage agreement for the SP model is shown on the x-axis, while the percentage agreement of the KVFD model is shown on the y-axis. The SP and KVFD models have mean percentage agreements of 94.91% and 96% with experimental data for each of the volunteers, respectively. Additionally, there was a significant Pearson correlation of 0.846 between the two models. Table 4 shows the best parameters for reconstructing healthy skin tissue for fifteen volunteers utilizing PIP between two models and a TWE sensor.

**Table 3:** Quantitative Errors in Fit Comparisons between SP and KVFD Models: This table lists the percentage agreement for SP and KVFD alongside the absolute differences, showcasing the error margins in the model fits for each volunteer across the studied frequency range

# of V	SP (%)	KVFD (%)	Error (%)
1	95.47	99.00	3.53
2	96.27	98.25	1.98
3	96.55	96.89	0.34
4	96.13	96.77	0.65
5	95.60	99.11	3.51
6	97.41	97.07	0.33
7	93.44	98.71	5.27
8	98.54	99.59	1.06
9	90.58	96.97	6.40
10	96.79	98.23	1.43
11	93.50	93.54	0.05
12	89.13	88.48	0.65
13	96.61	95.94	0.68
14	94.91	94.44	0.46
15	92.78	97.10	4.33

**Table 4:** Reconstruction of healthy skin parameters using the TOF method for SP and KVFD models

# of V	SP Model		KVFD Model		
	$\varvec{\eta }$ $(Pa\cdot s)$	$\varvec{\alpha }$	$\varvec{\eta }$ $(Pa\cdot s)$	$\varvec{\alpha }$	$\varvec{\mu }$ (Pa)
1	10.8	0.95	7.3	1.00	38840
2	9.5	0.95	7.2	1.00	29570
3	9.8	0.95	6.1	1.00	610
4	10	0.95	7.4	1.00	31830
5	8	0.95	9.1	0.95	23825
6	8.5	1.00	9.2	1.00	1625
7	8.3	0.95	8.3	0.95	30635
8	9.3	0.95	6.2	1.00	2230
9	9.5	0.95	5.4	1.00	42545
10	9.8	0.9	4.1	1.00	19860
11	8.5	1.00	9.3	1.00	1800
12	12	1.00	9.2	1.00	96860
13	10.8	0.95	8.5	1.00	29585
14	11	0.95	8.3	1.00	30285
15	8.8	0.95	8.6	0.95	34645

**Fig. 8:**
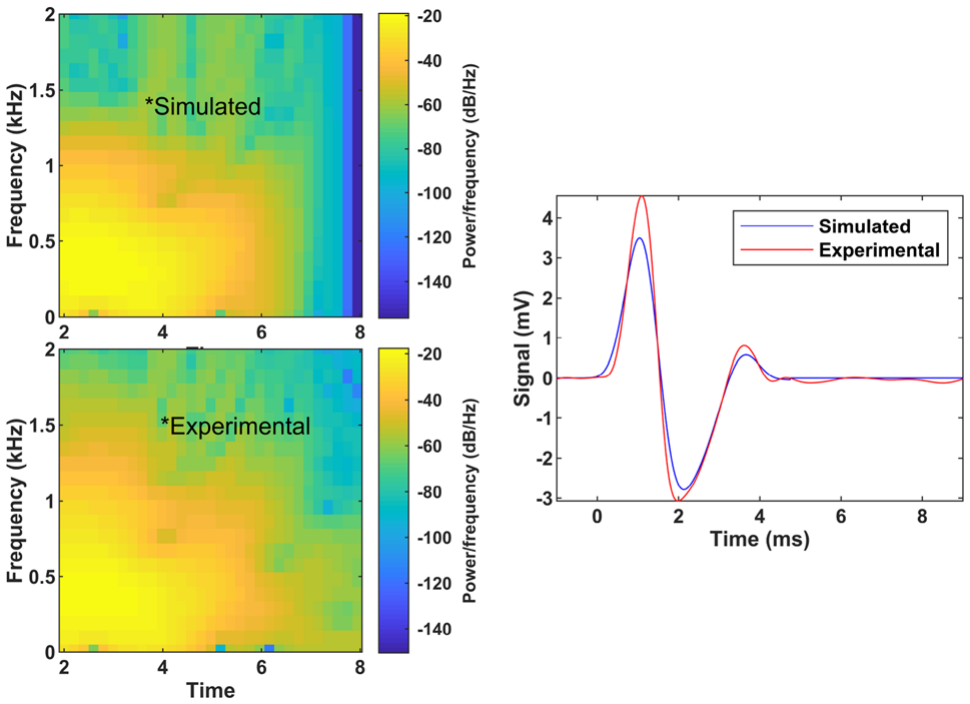
the Pearson correlation coefficient and the time-frequency plot for both the experimental and synthetic signals of volunteers 4

Figure 8 shows how the Pearson correlation and time-frequency plot fit the experimental and synthetic signals for Participant 4. The Pearson correlation coefficient between the two signals is 0.99. After resolving the PIP, which involves comparing the experimental signals (obtained via TWE measurements) with those generated by each model, the derived parameters are listed in Table 5.

**Table 5:** Reconstruction of healthy skin parameters Using FSW Method for SP and KVFD models

# of V	SP Model		KVFD Model		
	$\varvec{\eta }$ $(Pa \cdot s)$	$\varvec{\alpha }$	$\varvec{\eta }$ $(Pa \cdot s)$	$\varvec{\alpha }$	$\varvec{\mu }$ (Pa)
1	8.2	1.00	9.4	1.00	49720
2	12	0.95	8.5	1.00	45290
3	7.8	1.00	9.1	1.00	32985
4	7.7	1.00	9.2	1.00	42040
5	10.9	0.95	7.3	1.00	38930
6	10.8	1.00	9.5	1.00	74280
7	11	0.95	6.5	1.00	46455
8	12	0.95	9.3	1.00	26020
9	12	0.95	6.3	1.00	56640
10	8.3	0.95	8.3	0.95	28475
11	10.8	1.00	9.3	1.00	74180
12	12	1.00	9.1	1.00	122190
13	8.3	1.00	9.2	1.00	47965
14	8.5	1.00	9.3	1.00	49335
15	11.5	0.95	9.2	0.95	49620

### Comparative analysis of KVFD and SP rheological models based on TOF and FSW parameters reconstruction

The theoretical equations used in this study, with a focus on the SP model and the KVFD model, provide fundamental insights into the mechanical properties of skin tissue. Theoretically, the SP model estimated skin tissue viscosity ($\eta$) values ranging from 7.5 to 11.5 $Pa\cdot s$. In contrast, the KVFD model offered a wider range for $\eta$, with values ranging from 3.3 to 9.3 $Pa \cdot s$, and additionally suggested a range for ($\mu$) from 1.44 to 30.07 kPa. The main focus of the study’s results is on the reconstruction parameters that were found from the TOF and FSW for the SP model and the KVFD model in terms of the properties of skin tissue. For TOF and the SP model, the viscosity ($\eta$) values for skin tissue were found to be in the range of 8 to 12 $Pa \cdot s$. KVFD model showed a slightly lower range for $\eta$, with values varying from 4.1 to 9.3 $Pa \cdot s$. In addition, KVFD model showed a range of ($\mu$) values from 0.61 to 96.86 kPa for skin tissue. In addition, the FSW parameters indicated that, in the context of the SP model, the $\eta$ values for skin tissue ranged from 7.8 to 12 $Pa \cdot s$. For the KVFD model, the $\eta$ values were found to range from 6.3 to 9.5 $Pa \cdot s$, and the $\mu$ values were between 26.02 and 122.19 kPa for skin tissue. Furthermore, it was observed that the KVFD model converges to a simpler 2-parameter Kelvin-Voigt (KV) model when $\alpha$ parameter values are near 1. A comparison of the values of the $\alpha$ parameter between the SP model and the KVFD model showed that the reconstructions typically have lower values in the SP model than in the KVFD model.

## Discussion

The examination of human skin from a mechanical perspective poses considerable challenges across multiple disciplines. The quantification of the mechanical properties of human skin plays a crucial role in the identification and diagnosis of various skin diseases. Additionally, it improves in the assessment of the effectiveness of dermatological products [51].

Rheometry techniques are often regarded as the most precise means of quantifying the viscoelastic characteristics exhibited by soft tissues. However, these techniques are limited to the application on materials that are cultured in a laboratory setting (*in vitro *) or removed from the living organism for experimentation (*ex vivo *). The utilization of dynamic mechanical analysis (DMA) for the *in vitro * characterisation of soft tissue is subject to several constraints. Nevertheless, certain *in vivo * methodologies have the capability to autonomously evaluate the viscosity of tissues [52].Furthermore, the study was unable to conduct higher frequency measurements in DMA because to the inertial restrictions imposed by the rheometer utilized. In the previous study, the Dynamic Mechanical Analysis (DMA) experiments were carried out using a minimum frequency of 4 Hz, while the upper limit of the used frequency was below 50 Hz [52]. Consequently, the task of comparing the two techniques across diverse samples and with a high frequency has significant challenges.

TWE emerges as an established modern alternative to dynamic elastography, with notable advantages. The TWE method is a technique utilized in the assessment of the viscoelastic characteristics of skin tissue. The present technique is centered on the transmission of shear waves within biological tissue. Hence, its applicability extends not only in terms of depth but also in the radial direction, making it suitable for many applications including skin tissue. Axisymmetric waves facilitate the meticulous examination of the mechanical properties of skin tissues within cylindrical geometries. This study demonstrates the capability of the TWE technology to transmit and receive shear waves within the frequency range of 400 Hz to 1 kHz. In comparison to elastography devices that are commercially accessible, there exists a distinct benefit within this particular frequency band. The KV model, along with its fractional derivative counterpart, has demonstrated effective applicability in accurately modeling cervical rheometry and TWE data across a wide range of frequencies. [8, 53].

The team at the Ultrasonics Lab created and produced the TWE sensor, which underwent testing on 15 healthy volunteers. The observed dispersion curves of the volunteers exhibit a discernible pattern wherein the velocity of the waves demonstrates either a rise or decrease in relation to the frequency. This finding suggests that the skin tissue possesses viscoelastic properties, which aligns with the existing body of scholarly research on the subject. [54]. The reconstruction of skin parameters using the PIP involves three distinct methods: the theoretical, the TOF, and FSW. Theoretical estimates of skin tissue viscosity ($\eta$) show agreement between the SP and KVFD models. The SP model’s range of 7.5 to 11.5 $Pa \cdot s$, although slightly narrower, highlights its focused applicability compared to the broader range of 3.3 to 9.3 $Pa \cdot s$ presented by the KVFD model. In particular, the KVFD model’s additional estimation of ($\mu$) provides a range from 1.44 to 30.07 kPa [55–58]. TOF measurements for the SP model yielded $\eta$ values ranging from 8 to 12 $Pa \cdot s$ [56, 57], while the KVFD model showed a slightly wider range (4.1 to 9.3 $Pa \cdot s$) [56–58]. This pattern is similarly observed in the FSW measurements, where the SP model indicated $\eta$ values from 7.8 to 12 $Pa \cdot s$ [56, 57] and the KVFD model showed a range of 6.3 to 9.5 $Pa \cdot s$ [56–58]. Notably, both methods consistently show higher range of ($\mu$) values in the KVFD model, compared to the literature, which could be due to small variations in the distance between emitter and receiver while measuring. Both TOF and FSW methods have shown efficacy in our study. For the reconstruction of properties in a single layer, both methods exhibit comparable performance. However, when dealing with multi-layered reconstructions, the FSW method holds a distinct advantage. This is because the FSW approach allows for a more intricate analysis of wave reflections and the impact of adjacent layers on the properties under investigation. Therefore, in scenarios involving complex multi-layered structures, the FSW method might be preferable. An interesting observation is the convergence of the KVFD model to a simpler two-parameter Kelvin-Voigt (KV) model, especially when $\alpha$ parameter values are close to 1. This convergence suggests a flexibility within the KVFD model to adapt to a simpler form, potentially offering a balance between complexity and computational efficiency. However, it also highlights the inherent complexity of the KVFD model, which may not always be necessary or practical, especially in applications where simpler models are sufficient. A key advantage of the SP model is its simplicity and computational efficiency. With fewer parameters, the SP model is inherently simpler in algorithmic implementation and computational load. This simplicity is not only a matter of ease, but also translates into practical utility, particularly in scenarios where computational resources are limited or where rapid data processing is critical.

Therefore, based on the aforementioned study, it is feasible to determine the most effective technique or model. The SP model emerges as the superior choice even when additional considerations such as computational efficiency and complexity are taken into account.

However, the layered and anisotropic nature of the skin, although assumed to be isotropic in this study, could potentially have an effect on the results. This limitation is acknowledged and will be investigated in future research. The effect of gender and age on skin tissue biomarkers using this novel approach warrants further investigation. The SP model offers a precise mathematical depiction of the viscoelastic characteristics observed in skin tissue, as well as in complex tissues like cartilage or arterial walls. An examination of the changes in the mechanical properties of skin tissue can help with the detection of tumors in small organs like the mouth and glands.

## Conclusion

In this study, we applied PIP techniques and TWE rheological modeling to reconstruct skin parameters, using both SP and KVFD models for simulation. Our findings indicate that the SP model is the most suitable for simulating skin tissue behavior due to its accuracy and simplicity, requiring only two parameters compared to the KVFD model’s three. Experimental data were acquired from 15 volunteers using a TWE sensor, crucial for the reconstruction process. These data were then compared with numerical results. In our evaluations of the TOF and FSW methods, we found both to be effective for our study’s homogenous tissue model. However, we speculate that in multi-layer reconstructions, the FSW method could offer distinct advantages by providing a more nuanced analysis of wave reflections and their impacts on properties of adjacent layers. While our current research does not empirically demonstrate this in layered tissues, it contributes to dermatology by suggesting potential directions for effective modeling and reconstruction methods in skin property analysis, which may aid future clinical approaches.

## Data Availability

The datasets used and/or analysed during the current study available from the corresponding author on reasonable request.
